# The surgical outcome of standard lobectomy versus sleeve lobectomy in patients with non-small cell lung cancer: propensity score matching

**DOI:** 10.1093/icvts/ivae133

**Published:** 2024-07-13

**Authors:** Melike Ülker, Melek Ağkoç, Fahmin Amirov, Salih Duman, Berker Özkan, Mustafa Erelel, Murat Kara, Alper Toker

**Affiliations:** Department of Thoracic Surgery, Istanbul University, Istanbul Medical Faculty, Istanbul, Turkey; Department of Thoracic Surgery, Cerrahpasa University Medical Faculty, Istanbul, Turkey; Department of Thoracic Surgery, Azerbaijan Clinical Medical Center, Baku, Azerbaijan; Department of Thoracic Surgery, Istanbul University, Istanbul Medical Faculty, Istanbul, Turkey; Department of Thoracic Surgery, Istanbul University, Istanbul Medical Faculty, Istanbul, Turkey; Department of Pulmonary Diseases, Istanbul University, Istanbul Medical Faculty, Istanbul, Turkey; Department of Thoracic Surgery, Istanbul University, Istanbul Medical Faculty, Istanbul, Turkey; Department of Cardiovascular and Thoracic Surgery, West Virginia University Heart and Vascular Institute, Morgantown, WV, USA

**Keywords:** Lobectomy, Sleeve resection, Non-small-cell lung cancer, Complication, Mortality

## Abstract

**OBJECTIVES:**

The goal of this study was to compare the patients who underwent standard or sleeve lobectomy for non-small cell lung cancer in terms of postoperative outcomes, prognostic factors and overall survival.

**METHODS:**

Between January 2002 and January 2020, the patients with squamous cell carcinoma or adenocarcinoma who underwent standard lobectomy or sleeve lobectomy by thoracotomy in our clinic were analysed retrospectively. Standard and sleeve groups were compared after propensity score matching in terms of age, comorbidity, T status, N status and pathological stage. Primary outcomes were morbidity and mortality; the secondary outcome was overall survival.

**RESULTS:**

The study included 476 patients, and sleeve lobectomy was performed in 196 (41.1%) patients. Multivariable analysis revealed that age over 61 years (*P* = 0.003 and *P* = 0.005, respectively), forced expiratory volume in 1 s (FEV1) below 84% (*P* = 0.013 and *P* = 0.205, respectively) and the presence of perineural invasion (*P* = 0.052 and *P* = 0.001, respectively) were poor prognostic factors in the standard lobectomy and the sleeve groups. The propensity matching analysis included 276 patients (138 sleeve lobectomy and 138 standard lobectomy). Complications occurred in 96 (69.6%) and 92 (66.7%) patients in the standard and sleeve groups, respectively (*P* = 0.605). Three (2.2%) patients in the standard group and 5 (3.6%) patients in the sleeve group died within 90 days postoperatively (*P* = 0.723).

**CONCLUSIONS:**

Bronchial sleeve lobectomy is a safe procedure that can be applied in oncologically suitable cases without causing higher mortality than a standard lobectomy.

## Graphical Abstract

**Figure:**
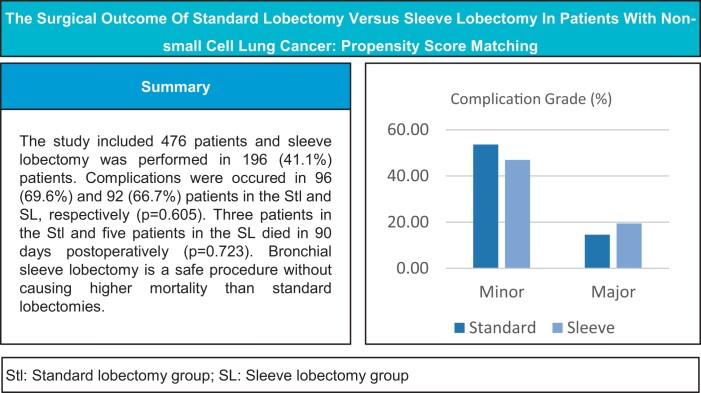

## INTRODUCTION

Lung cancer is one of the most common types of cancers worldwide, and deaths due to lung cancer rank first among all cancer-related deaths. In our country, lung cancer is the most common cause of death due to cancer with a rate of 17% [1]. Its prevalence is increasing, especially in developing countries. Despite developing technological possibilities, advancements in surgical techniques and in non-surgical treatment methods, 5-year survival rates for lung cancer remain at approximately 15% [2].

For many years, pneumonectomy was considered the only acceptable type of surgery, especially for centrally located or locally advanced lung tumours. Bronchoplastic resections such as bronchial sleeve lobectomy have been developed as an alternative to pneumonectomy, which is a radical resection with a parenchyma-sparing aspect. The bronchial sleeve lobectomy was first introduced as a parenchyma-sparing operation by Sir Clement Price-Thomas in 1947, and in 1954 Allison performed the first sleeve lobectomy for bronchogenic carcinoma. Bronchoplastic procedures were initially used in patients with impaired lung function but are now also favoured in anatomically suitable patients [3, 4].

The frequency of sleeve lobectomy has increased with the advances in surgical techniques and successful results. In cases of primary lung carcinoma, the rate of bronchial sleeve lobectomy has increased from 3.4% to 13% [5, 6]. In this study, our goal was to explain how sleeve lobectomy may pose additional perioperative and postoperative risks compared to standard lobectomy, based on our own clinical experience.

## MATERIALS AND METHODS

### Patient selection

Between January 2002 and January 2020, patients who underwent sleeve or standard lobectomy by thoracotomy for non-small cell lung cancer in the Department of Thoracic Surgery, Istanbul University, Istanbul Faculty of Medicine, were retrospectively analysed through the data bank records. The data of patients who were operated on by a single surgical team for adenocarcinoma and squamous cell carcinoma were analysed. Demographic characteristics, postoperative complications, drainage time, hospital stay, mortality, pathological staging, histopathological type, survival and prognostic factors were determined. A comparative analysis was performed between patients who underwent a standard or a sleeve lobectomy. The primary outcomes were morbidity and mortality; the secondary outcome was overall survival.

### Exclusion criteria

Patients with benign or secondary lung malignancy, patients with a diagnosis other than squamous cell carcinoma and adenocarcinoma on pathological examination, patients with positive surgical margins, patients with known metastases preoperatively or metastases detected on pleural or pericardial surfaces by postoperative pathological analysis and patients with multiple N2 lymph node involvement on postoperative pathological analysis were excluded from the study.

### Preoperative evaluation

Blood biochemistry tests, pulmonary function tests and arterial blood gas analyses were performed preoperatively. All patients were prepared for surgery after thorax computed tomography (CT), positron emission tomography/computed tomography (PET/CT), cranial magnetic resonance imaging for distant metastasis screening and clinical staging.

### Preoperative invasive staging

Cervical standard mediastinoscopy was performed for invasive mediastinal staging. In our clinic, mediastinoscopy in primary lung cancer is performed in cases in which clinical staging indicates lymph node metastasis, such as the presence of mediastinal lymph nodes larger than 1 cm on thoracic CT or suspected lymph node involvement in the mediastinum on PET/CT.

### Operative approach

Thoracotomy was the preferred method, based on the location of the lesion and the type of operation planned. Sleeve lobectomy was preferred in the presence of tumour fixed to the lobe bronchus or lymph node invasion. In sleeve lobectomies, both (distal and proximal) bronchial surgical margins were examined histopathologically by frozen section before the bronchial anastomosis. In sleeve lobectomies, bronchial anastomoses were supported with intercostal muscle flaps, pericardium or parietal pleura or were left unsupported.

### Postoperative follow-up

Cardiac arrhythmia, a prolonged air leak exceeding 5 days, pneumonia (antibiotics change when infection parameters do not decrease sufficiently or when pneumonia is suspected from the chest radiograph), atelectasis requiring bronchoscopy, bronchopleural fistula, empyema, development of bronchial stenosis at the operation site, haemorrhage requiring revision, chylothorax, myocardial infarction and a cerebrovascular ischaemic attack were determined and recorded as complications. Postoperative pathological staging was performed according to the 8th TNM classification. Patients were followed up in thoracic surgery and oncology outpatient clinics after discharge. In the first year after the operation, they were evaluated with a computed tomography every 3 months and, after the second year, every 6 months.

### Statistical evaluation

IBM SPSS Statistics Version 26 (IBM-SPSS Inc., Armonk, NY, USA) and a propensity score program were used. Patients were divided into a standard and a sleeve lobectomy group. The Pearson χ^2^ test and the Fisher exact test were used for categorical variables, and the Mann–Whitney *U* test was used for non-categorical variables. A *P*-value below 0.05 was considered significant. Survival curves were plotted using the Kaplan–Meier method. The log-rank test was used to compare survival times between the different groups. For parameters with a *P*-value <0.1, multivariate analyses were performed using Cox-regression tests.

Propensity score matching with a logistic regression model was used in standard and sleeve lobectomy groups. Age, presence of comorbidity, T stage (T1–2–3–4), N stage (N0–1–2), histopathological diagnosis (squamous cell carcinoma, adenocarcinoma), and pathological staging (stages I–II–III–IV) were taken into consideration and compared using the closest values in a matched patient sample for all patients.

The study was approved by the ethics committee of Istanbul University, Istanbul Faculty of Medicine and the ethics committee number is 2020/1444.

## RESULTS

### Demographic characteristics

The study included 476 patients who underwent standard lobectomy or sleeve lobectomy. Of these patients, 280 underwent standard lobectomy and 196 underwent sleeve lobectomy. Demographic characteristics of the patients are shown in Table 1.

**Table 1:** Demographic features of the standard lobectomy versus the sleeve lobectomy groups before and after propensity score matching

	Before propensity score matching			After propensity score matching		
	Standard lobectomy	Sleeve lobectomy	*P*-value	Standard lobectomy	Sleeve lobectomy	*P*-value
	*n* = 280 (%)	*n* = 196 (%)				
				*n* = 138 (%)	*n* = 138 (%)	
Age (median ± SD)	60.5 ± 9.2	61 ± 8.9	0.446	61 ± 9.1	60.8 ± 9.2	0.931
Female	68 (24.3)	16 (8.2)	**< 0.001**	29 (21)	10 (7.2)	**0.001**
Male	212 (75.7)	180 (91.8)		109 (79)	128 (92.8)	
Left lung	111 (39.6)	62 (31.6)	0.074	50 (36.2)	44 (31.9)	0.446
Right lung	169 (60.4)	134 (68.4)		88 (63.8)	94 (68.1)	
Smoking history	225 (80.4)	174 (88.8)	**0.014**	111 (80.4)	118 (85.5)	0.262
Comorbidity	169 (60.4)	109 (55.6)	0.301	83 (60.1)	73 (52.9)	0.225
Respiratory functions (mean ± SD)						
FEV1 (ml)	2387 ± 614	2351 ± 663	0.587	2382 ± 618	2411 ± 673	0.709
FEV1 (%)	86 ± 18	80 ± 18	**0.001**	84 ± 17	81 ± 18	0.088
FVC (ml)	3190 ± 775	3198 ± 785	0.693	3209 ± 755	3292 ± 790	0.359
FVC (%)	92 ± 17	87 ± 18	**0.001**	91 ± 15	88 ± 17	0.092
DLCO	83 ± 19	86 ± 20	0.211	83 ± 14	84 ± 13	0.488
Histopathological analysis						
Squamous cell carcinoma	129 (46.1)	158 (80.6)	**< 0.001**	96 (69.6)	100 (72.5)	0.596
Adenocarcinoma	151 (53.9)	38 (19.4)		42 (30.4)	38 (27.5)	
T status						
T1	56 (20)	58 (29.6)		30 (21.7)	32 (23.2)	
T2	76 (27.1)	76 (38.8)	**< 0.001**	51 (37)	47 (34.1)	0.964
T3	101 (36.1)	42 (21.4)		38 (27.5)	40 (29)	
T4	47 (16.8)	20 (10.2)		19 (13.8)	19 (13.8)	
N status						
N0	187 (66.8)	100 (51)		82 (59.4)	73 (52.9)	
N1	63 (22.5)	78 (39.8)	**< 0.001**	37 (26.8)	53 (38.4)	0.084
N2	30 (10.7)	18 (9.2)		19 (13.8)	12 (8.7)	
Pathological stage						
I	72 (25.7)	56 (28.6)		40 (29)	32 (23.2)	
II	112 (40)	83 (42.3)	0.168	53 (38.4)	58 (42)	0.129
III	90 (32.1)	57 (29.1)		41 (29.4)	48 (34.8)	
IV	6 (2.1)			4 (2.9)		
Drainage time (days)	6 ± 7.6	6 ± 9.9	0.201	6 ± 5.5	6 ± 9.6	0.665
Hospital stay (days)	9 ± 10.7	10 ± 11.8	**0.017**	9 ± 8.2	9.5 ± 12	0.271
Complications	189 (67.5)	132 (67.3)	0.972	96 (69.6)	92 (66.7)	0.605
Death (90 days)	6 (2.1)	9 (4.5)	0.132	3 (2.2)	5 (3.6)	0.723
Survival (5 years)	58%	53%	0.398	55%	49%	0.482

DLCO: diffusion capacity for carbon monoxide; FEV1: forced expiratory volume in first second; FVC: forced vital capacity; N: node; SD: standard deviation; T: tumour.

*P* < 0.05 values were bolded.

In the sleeve resection group, the bronchial sleeve was performed in 158 (80.6%) cases; the bronchial sleeve and pulmonary artery patch plasty were applied in 18 (9.2%) cases; and double sleeves were applied in 20 (11.2%) cases.

Four of the stage IV patients included in the study had oligometastatic diseases, of which 3 were brain metastases and 1 was an adrenal gland metastasis. In 2 cases, metastases were detected in the postoperative pathological examination of a sample taken from the pleural fluid detected perioperatively.

### Complications

Complications detected in the standard and sleeve lobectomy groups are shown in Table 2. Complication rates were 67.5% (*n* = 189) and 67.3% (*n* = 132) in standard and sleeve lobectomy groups, respectively. There was no significant difference in the incidence of complications between the groups (*P* = 0.972). Complications grouped according to the Clavien-Dindo classification [7] are shown in Table 3. There was no statistically significant difference between the groups in terms of complication score (*P* = 0.261).

**Table 2:** Postoperative complications of standard and sleeve lobectomy groups

Complications	Standard lobectomy		Sleeve lobectomy	
	*n*	%	*n*	%
Prolonged air leak	99	35.4	63	32.1
Arrhythmia/atrial fibrillation	32	11.4	44	22.4
Pneumonia	58	20.7	41	20.9
Empyema	5	1.7	5	2.5
Wound infection	6	2.1	7	3.5
Bronchopleural fistula	–	–	3	1.5
Chylothorax	7	2.5	4	2
Respiratory failure	20	7.1	2	1
Blood transfusion	43	15.3	8	4
Acute kidney failure	5	1.7	3	1.5
Myocardial infarction	3	1	3	1.5
Other	14	5	6	3
*P*-value	0.972			

**Table 3:** The Clavien-Dindo classification of surgical complications

Complication grade	Standard lobectomy	Sleeve lobectomy
	*n *=* *191	*n *=* *130
**Minor**	**150 (53.6%)**	**92 (46.9%)**
Grade I	38 (13.6%)	26 (13.3%)
Grade II	112 (40%)	66 (33.7%)
**Major**	**41 (14.6%)**	**38 (19.4%)**
Grade IIIa	12 (4.3%)	17 (8.7%)
Grade IIIb	20 (7.1%)	10 (5.1%)
Grade IV	3 (1.1%)	2 (1%)
Grade V	6 (2.1%)	9 (4.6%)
*P*-value	0.261	

The minor and major distinctions are bolded to ensure easy visibility.

The incidence of complications was compared between the subgroups of median age over 61 years, smoking status, presence of comorbidity, neoadjuvant treatment, T stage and N stage (Table 4). In the standard lobectomy group, advanced T stage was found to be significantly associated with the development of complications (*P* < 0.0001). In the sleeve lobectomy group, being over 61 years of age (*P* = 0.005) and receiving neoadjuvant treatment (*P* = 0.012) were found to be significant in terms of the development of complications.

**Table 4:** Occurrence of complications in standard and sleeve lobectomy groups

	Occurrence of complications			
	Standard lobectomy	*P*-value	Sleeve lobectomy	*P*-value
Age				
≤61	95/140 (67%)	0.898	54/94 (57%)	**0.005**
>61	94/140 (67%)		78/102 (76%)	
Smoking				
Yes	157/225 (69%)	0.100	116/174 (66%)	0.568
No	32/55 (58%)		16/22 (72%)	
Neoadjuvant therapy				
Yes	56/75 (74%)	0.121	38/46 (82%)	**0.012**
No	133/205 (64%)		94/150 (62%)	
Comorbidities				
Yes	118/169 (69%)	0.306	77/109 (70%)	0.271
No	71/111 (63%)		55/87 (63%)	
T status				
T1–T2	75/132 (56%)	**<0.0001**	90/134 (67%)	0.936
T3–T4	114/148 (77%)		42/62 (67%)	
N status				
N0	128/187 (68%)	0.631	67/100 (67%)	0.916
N1–N2	61/93 (65%)		65/96 (67%)	

N: node; T: tumour.

Values with *P* < 0.05 have been bolded.

### Postoperative period and mortality

The median length of hospital stay was 9 days [standard deviation (SD): 11.2 (range: 3–108)], whereas it was 9 ± 10.7 days in the standard group and 10 (SD: 11.8) days in the sleeve lobectomy group (*P* = 0.017). There was no significant difference between the groups in terms of neoadjuvant treatment rate (*P* = 0.413), whereas the hospital stay was 12.1 (SD: 10.8) days in patients who did not receive neoadjuvant treatment and 14.2 (SD: 12.2) days in those who received neoadjuvant treatment (*P* = 0.070).

Postoperative mortality (90 days) was observed in 6 (2.1%) cases in the standard lobectomy group and in 9 (4.5%) cases in the sleeve lobectomy group. Age, forced expiratory volume in 1 s (FEV1) percentage, T status (early/advanced), N status (N0/1/2), stage (early/advanced), presence of prognostic factors and presence of perineural invasion were included in the multivariable analysis of the parameters with significant *P-*values in the univariate analysis (Table 5) in terms of postoperative mortality between both groups.

**Table 5:** Univariable prognostic factor analysis with Cox-regression in the standard and sleeve lobectomy groups

	Univariable analysis					
	Standard lobectomy			Sleeve lobectomy		
Variables	HR	95% CI (lower–upper)	*P*-value	HR	95% CI (lower–upper)	*P*-value
Age						
61>/61≤	1.882	1.307–2.710	**0.001**	1.778	1.195–2.645	**0.005**
Gender						
Female/male	1.147	0.753–1.746	0.524	1.122	0.566–2.226	0.742
FEV1 (ml)						
2372 ≤/2372 >	1.335	0.937–1.902	0.110	1.349	0.910–1.998	0.136
FEV1 (%)						
84 ≤/84 >	1.598	1.123–2.274	**0.009**	1.271	0.849–1.904	0.244
Smoking history						
Present/absent	1.135	0.727–1.773	0.577	1.170	0.640–2.140	0.610
Location						
Left/right	1.027	0.859–1.228	0.769	1.078	0.878–1.325	0.473
Neoadjuvant therapy						
Absent/present	1.462	1.001–2.135	0.050	1.261	0.806–1.972	0.309
Histopathological analysis						
Adenocarcinoma/SCC	1.147	0.807–1.630	0.445	1.097	0.678–1.773	0.706
T status						
T3–4/T1–2	1.383	0.970–1.971	**0.073**	1.067	0.699–1.630	0.763
N1	1.060	0.688–1.631	0.792	1.415	*0.933–2.145*	0.102
N2	1.925	1.152–3.216	**0.012**	2.635	*1.412–4.916*	**0.002**
Pathological stage						
Stage III–IV/stage I–II	1.589	1.110–2.276	**0.011**	1.922	1.280–2.888	**0.002**
Prognostic factor						
Present/absent	1.477	0.952–2.289	**0.082**	1.269	0.808–1.995	0.301
Stromal infiltration						
Present/absent	1.069	0.749–1.526	0.712	1.038	0.697–1.546	0.853
Lymphatic invasion						
Present/absent	1.470	1.034–2.091	0.032	1.050	0.709–1.557	0.806
Vascular invasion						
Present/absent	1.318	0.910–1.909	0.144	1.290	0.808–2.061	0.286
Perineural invasion						
Present/absent	1.675	1.065–2.635	**0.026**	1.993	1.271–3.124	**0.003**

**CI:** confidence interval; FEV1: forced expiratory volume in 1 s; N: node; SCC: squamous cell carcinoma.

Values with *P* < 0.05 have been bolded.

In the multivariable analysis (Table 6), in the standard lobectomy group, median age above 61 years [hazard ratio (HR) (95% confidence interval (CI): 1.755 (1.207—2.552), *P* = 0.003], FEV1 percentage below 84 [HR (95% CI): 1. 566 (1.098—2.234), *P* = 0.013] and the presence of perineural invasion [HR (95% CI): 1.599 (0.995—2.569), *P* = 0.052] were found to be poor prognostic factors.

**Table 6:** Multivariable prognostic factor analysis with Cox-regression in the standard and sleeve lobectomy groups

	Multivariable analysis					
	Standard lobectomy			Sleeve lobectomy		
Variables	HR	95% CI (lower–upper)	*P*-value	HR	95% CI (lower–upper)	*P*-value
Age						
61>/61≤	1.755	1.207–2.552	**0.003**	1.803	1.195–2.720	**0.005**
FEV1 (ml)						
84 ≤/84 >	1.566	1.098–2.234	**0.013**	1.303	0.865–1.962	0.205
T status						
T3–4/T1–2	1.260	0.815–1.948	0.298	1.001	0.584–1.715	0.997
N0/N1/N2	1.185	0.860–1.631	0.300	1.299	0.877–1.923	0.191
Pathological stage						
Stage III–IV/stage I–II	1.134	0.676–1.904	0.633	1.632	0.871–3.059	0.126
Prognostic factor						
Present/absent	1.194	0.758–1.882	0.445	1.470	1.139–1.899	**0.003**
Perineural invasion						
Present/absent	1.599	0.995–2.569	**0.052**	2.360	1.446–3.851	**0.001**

CI: confidence interval; FEV1: forced expiratory volume in 1 s; HR: hazard ratio; N: node; T: tumour.

Values with *P* < 0.05 have been bolded.

In the multivariable analysis, age above 61 years in the sleeve lobectomy group [HR (95% CI): 1.803 (1.195—2.720), *P* = 0.005], the presence of prognostic factors [HR (95% CI): 1.470 (1.139–1.899), *P* = 0.003] and the presence of perineural invasion [HR (95% CI): 2.360 (1.446–3.851), *P* = 0.001] were found to be poor prognostic factors.

In the postoperative period, 13 (4.6%) cases in the standard lobectomy group and 10 (5.1%) cases in the sleeve lobectomy group underwent revision with re-thoracotomy due to air leaks, haematoma, empyema, wound infections, chylothoraxes and bronchopleural fistulas.

### Propensity score matching

A total of 138 cases were selected from the standard lobectomy group compared to 138 in the sleeve lobectomy group. Demographic characteristics of the cases are shown in Table 1. According to propensity score matching, no significant differences occurred between the standard lobectomy and sleeve lobectomy groups in terms of complication development (*P* = 0.605) and postoperative mortality (*P* = 0.723).

**Figure 1:**
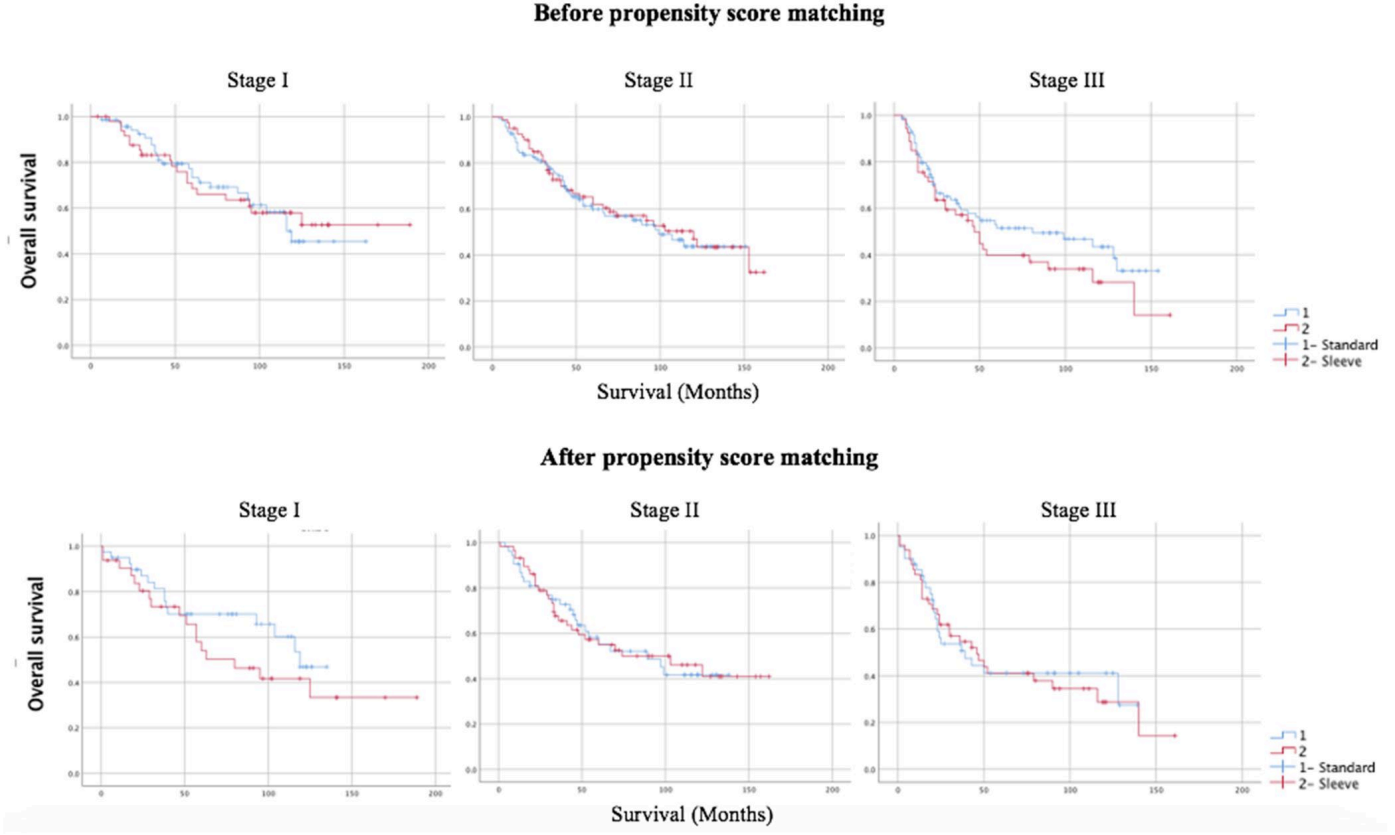
Survival analysis of stages I, II and III patients in groups undergoing standard and sleeve lobectomies before and after propensity score matching.

When the 5-year survival rates before propensity score matching were evaluated, they were found to be 70%, 59% and 51% in the standard lobectomy group and 68%, 62% and 44% in the sleeve group in stage 1, stage 2 and stage 3, respectively (Fig. 1).

After propensity score matching, the 5-year survival rate was 55% in the standard group and 49% in the sleeve lobectomy group (*P* = 0.482).

## DISCUSSION

The frequency of sleeve lobectomy has increased with advances in surgical techniques and successful results. In cases of primary lung carcinoma, the rate of bronchial sleeve lobectomy has increased from 3.4% to 13% [5, 6].

When comparing patients receiving neoadjuvant treatment, complications were seen in 74% of those who received neoadjuvant treatment in the standard group and 82% of those who received neoadjuvant treatment in the sleeve lobectomy group. Although no increased risk of complications was associated with neoadjuvant treatment in the conventional group, an increased risk was noted in the sleeve lobectomy group. Bagan *et al.,* in 2009, showed that neoadjuvant treatment was not a risk factor for increased mortality and morbidity in patients who underwent sleeve resection. In previous studies, an air leak was defined as 7 days or more, whereas we accepted 5 days or more, so there is a difference in the rate reported in the literature and that reported in our study. In addition to the newly developed induction chemotherapy drugs and schemes, we also observed that postoperative mortality decreased despite postoperative complications with the completion of the learning curve [5].

The most common complications in our study were prolonged air leaks (PAL) and cardiac arrhythmias. In the standard and sleeve lobectomy groups, PAL rates were 35.4% and 32.1%, respectively, whereas cardiac arrhythmia rates were 11.4% and 22.4%. There was no significant difference in the development of PAL in sleeve resections, but the development of cardiac arrhythmias was found to be increased. In the study by Melloul *et al.* (2009), increased pulmonary complications were noted in the sleeve lobectomy group [8].

We found no significant difference between the 2 groups in terms of complication development as a result of propensity score matching. In the study by İnci *et al*., no significant difference was found between the standard and sleeve resection groups in terms of the development of pulmonary and cardiac complications [9].

In the literature, mortality rates for sleeve lobectomy range from 0% to 7.5% [10, 11]. In a meta-analysis of patients who underwent sleeve resection, in 2014, mortality rates were shown to be between 0% and 4.5% [12]. In our study, postoperative mortality was 3.1% in the whole group and 2.1% and 4.5% in the standard and sleeve groups, respectively, which was acceptable according to the literature. There was no significant difference in mortality between the 2 groups, and, after propensity score matching, no significant difference was found in mortality rates of 2.2% and 3.6% in the standard and sleeve lobectomy groups, respectively. In the study by İnci *et al*., no significant difference in mortality was shown between the 2 groups [9].

When survival analyses were examined, the 5-year survival rates varied between 37.5% and 72.5% in sleeve lobectomies [10, 11]. In our study, 5-year survival rates in the standard and sleeve groups were 60% and 57%, respectively, with no significant difference between the groups. Five-year survival analyses of propensity score matching according to stage, T and N status showed 55% in the standard lobectomy group and 49% in the sleeve lobectomy group. In the study by İnci *et al.,* the 5-year survival rate in the conventional and the sleeve groups was 57%, which is close to our results [8]. D’Andrilli *et al.,* in 2016, reported that the 5-year survival in the conventional and sleeve lobectomy group was 58% and 88%, respectively [13].

Gonzalez *et al.* in 2022 observed that a fistula developed in 2.3% of sleeve cases [14]. In our series, a bronchial fistula was observed in 3 cases (1.5%), 2 of which were re-operated on for revision.

In the presence of perineural invasion, the 5-year survival rates in the standard and sleeve groups were 47% and 32%, respectively, whereas the 5-year survival rates in the absence of perineural invasion were 62% and 64%, respectively. Within the groups, a significant survival difference was observed in the presence of perineural invasion.

When the parameters of age, FEV1 percentage, N status, pathological stage, presence of prognostic factors and presence of perineural invasion, for which we obtained significant results in our univariable analyses, were subjected to multivariable analysis, age below 61 and FEV1 percentage above 84% in the standard group were evaluated as factors positively affecting survival. In the sleeve group, age > 61 years and the presence of prognostic factors were evaluated as factors negatively affecting survival. In both groups, the presence of perineural invasion was found to have a significant and poor effect on survival.

### Limitations

Due to the increasing use of thoracoscopy, the use of thoracotomy in standard lobectomies has decreased, which has led to limitations in the patient group selected for the study. Local recurrence could not be evaluated because the patients received postoperative oncological treatment processes in different centres.

## CONCLUSION

Bronchial sleeve lobectomy is a procedure that can be safely performed in oncologically suitable cases without a higher mortality rate than standard lobectomy. In non-small cell lung cancer, sleeve lobectomy can be performed safely if it is surgically and oncologically appropriate.

## Data Availability

Statistical Package for the Social Sciences (SPSS) Statistics Version 26 and propensity score programs were used. The data in SPSS format can be sent upon request.

## Notes

Presented at the 29th European Conference on General Thoracic Surgery, 20–22 June 2021.

## ABBREVIATIONS

CI: Confidence interval
FEV1: Forced expiratory volume in 1 s
HR: Hazard ratio
SD: Standard deviation
